# Recombinase Polymerase Amplification assays for detection of the major tropical root-knot nematodes

**DOI:** 10.21307/jofnem-2021-109

**Published:** 2022-01-07

**Authors:** Sergei A. Subbotin, Julie Burbridge

**Affiliations:** 1 Plant Pest Diagnostic Center, California Department of Food and Agriculture, 3294 Meadowview Road, Sacramento, CA 95832

**Keywords:** Diagnostics, *Meloidogyne areanaria*, *Meloidogyne incognita*, *Meloidogyne javanica*, Recombinase polymerase amplification, Tropical root-knot nematode complex

## Abstract

Detection of root-knot nematodes (RKN) in soil and plant samples is crucial to prevent its spread and select effective control measures. In this study, Recombinase Polymerase Amplification (RPA) assays using lateral flow dipsticks (LF-RPA) and real-time fluorescence detection (real-time RPA) were developed to detect the RKN species from tropical complex using a group-specific primer-probe set and *Meloidogyne javanica* using a species-specific primer-probe set. The results of the real time RPA assays in series of crude nematode extracts showed reliable detection within 16 min with a sensitivity of 1/100 of a second-stage juvenile in a reaction tube. The results of the LF-RPA assays showed reliable detection within 30 min with a sensitivity of 1/20 to 1/100 of a second-stage juvenile and 1/10 of a female in a reaction tube. Real-time RPA and LF-RPA assays are highly specific and can identify their target DNA in mixtures with other nematodes and plant tissues. LF-RPA assay has great potential for diagnosing RKN in the lab, field or in areas with a minimal laboratory infrastructure.

The root-knot nematodes (RKN) of the genus *Meloidogyne* represent one of the most damaging and agriculturally important groups of plant-parasitic nematodes. *Meloidogyne incognita*, *M*. *javanica* and *M*. *arenaria* are three major tropical RKN species, which are globally distributed and polyphagous pests of many agricultural crops. These nematodes together with several other species are commonly referred to the tropical RKN complex (Álvarez-Ortega et al., 2019).

Early and rapid detection of RKN in soil and plant roots is important for the application of effective control measures and prevention of spread of these agricultural pests. Currently, the reliable diagnosis of RKN is based on molecular methods using sequencing of nuclear ribosomal RNA and mitochondrial genes, PCR with specific primers and other techniques (Subbotin et al., 2021). Recombinase Polymerase Amplification (RPA) is a relatively new isothermal methodology for amplifying DNA and uses a highly efficient displacement polymerase that amplifies a few copies of target nucleic acid in several minutes at a constant temperature (37–42°C) (Daher et al., 2016; James and Macdonald, 2015; Piepenburg et al., 2006). RPA has several advantages over PCR-based methods of plant-parasitic nematode detection: (i) does not require thermal cycling and can be used in areas with minimal laboratory infrastructure and run by personals with minimal technical experience; (ii) in 10 or more times sensitive than PCR; (iii) does not require DNA extraction in sample processing; (iv) amplicons may be detected at endpoint or in real-time during 8 to 30 min. Real-time RPA assay with using fluorescent probes in real time for detection of plant-parasitic nematodes was first designed and published by Subbotin (2019) for *M. enterolobii*. Recently, RPA assays with a gel visualization of amplicons for species diagnostics of *M. javanica, M. arenaria, M. incognita* and *M. enterolobii* have also been developed by Ju et al. (2019). Chi et al. (2020) designed a rapid RPA assay for visual detection of *M. javanica* using SYBR Green and lateral flow dipsticks (LF-RPA) at endpoint. RPA assays for detection of the northern RKN, *M. hapla* were also developed by Song et al. (2021) and Subbotin and Burbridge (2021).

The goal of this study was to develop real-time RPA and LF-RPA assays with group-specific primers-probe sets for the detection of the major RKN belonging to the tropical RKN complex (*M. arenaria, M. floridensis, M. hispanica, M. incognita, M. javanica* and *M. paranaensis*) and species-specific primers-probe set the detection of *M. javanica* only.

## Materials and methods

### Nematode samples

Twenty six isolates of *M. javanica,* seven isolates of *M. arenaria,* six isolates of *M. incognita,* two isolates of *M. floridensis* and by one isolate of *M. hispanica* and *M. paranaensis* were used for RPA assay development and validation. DNA of several other RKN, *M. artiellia*, *M. baetica, M. christiei, M. enterelobii, M. hapla, M. haplanaria, M. naasi* and *M. nataliei* were also used in specificity experiments (Table 1). Second stage juveniles (J2s), eggs and females were extracted from root or soil samples. The *nad5* gene was amplified and then sequenced from each isolate to confirm its identity. Free-living and plant-parasitic nematodes from several field samples collected in California were extracted using the centrifugal flotation method and their extracts were used as background non-target DNA.

**Table 1.** Samples of the root-knot nematodes tested in the present study.

Species	Location	Plant	Sample code	Javanica specific RPA assay	Tropical complex specific RPA assay	Source
*Meloidogyne arenaria*	USA, Florida, Polk County	Peach	CD3093; N16-01563-3B	‒	+	J.A. Brito
*M. arenaria*	USA, Florida	Peach	CD2131, N16-00537-2	‒	+	J.A. Brito
*M. arenaria*	USA, Florida, Polk County	Peach	CD2213; N16-00026-2	‒	+	J.A. Brito
*M. arenaria*	USA, Florida, Levy County	Peanut	CD3100; MA0101-UF	‒	+	J.A. Brito
*M. arenaria*	USA, Florida, Alachua County	Water oak	CD3342; N20-00865-1	‒	+	J.A. Brito
*M. arenaria*	USA, Florida, Polk County	Curry tree	CD3343; N20-00676-1	‒	+	J.A. Brito
*M. arenaria*	USA, Florida, Polk County	Mixed roots	CD3344; N20-00678-6	‒	+	J.A. Brito
*M. artiellia*	Spain	Mixed roots	CD3475	‒	‒	P. Castillo
*M. baetica*	Spain	Olive	CD3382	‒	‒	P. Castillo
*M. christiei*	USA Florida	Turkey oak	CD1471	‒	‒	J.A. Brito
*M. enterolobii*	USA, Florida, Dade County	Mixed roots	CD3335; N20-00838-29	‒	‒	J.A. Brito
*M. enterolobii*	USA, Florida, St. Lucie County	Mixed roots	CD3399; N20-01132-8	‒	‒	J.A. Brito
*M. floridensis*	USA, California	Almond	CD3111	‒	+	S.A. Subbotin
*M. floridensis*	USA, Florida, St.Lucie County	Peach	CD3095; N19-00287	‒	+	J.A. Brito
*M. hapla*	USA, California	Tomato	VW9	‒	‒	V. Williamson
*M. hapla*	USA, California	Tomato	C44	‒	‒	V. Williamson
*M. haplanaria*	USA, California	Pitcher plant	CD3446	‒	‒	S.A. Subbotin
*M. hispanica*	Spain	Mixed roots	CD3516	‒	+	P. Castillo
*M. incognita*	USA, UCR collection	Tomato	CD3038; Isolate 18	‒	+	P. Roberts
*M. incognita*	USA, UCR collection	Tomato	CD3034; Isolate 47	‒	+	P. Roberts
*M. incognita*	USA, UCR collection	Tomato	CD3037; Isolate 49	‒	+	P. Roberts
*M. incognita*	USA, UCR collection	Tomato	CD3031; isolate 20	‒	+	P. Roberts
*M. incognita*	USA, Florida, Palm Beach County	Snake plant	CD3336; N20-00859-6	‒	+	J.A. Brito
*M. incognita*	USA, Florida, Palm Beach County	Mixed roots	CD3337; N20-00588-13	‒	+	J.A. Brito
*M. javanica*	USA, Florida, Polk County	Peach	CD2009; N16-00032-2	+	+	J.A. Brito
*M. javanica*	USA, Florida, Polk County	Peach	CD2011; N16-00033	+	+	J.A. Brito
*M. javanica*	USA, Florida, Pasco County	Peach	CD2012; N16-00082-2	+	+	J.A. Brito
*M. javanica*	USA, Florida, Pasco County	Peach	CD2013; N16-0078-3	+	+	J.A. Brito
*M. javanica*	USA, Florida, Pasco County	Peach	CD2016; N16-00082-1	+	+	J.A. Brito
*M. javanica*	USA, Florida, Pasco County	Peach	CD2059; N16-00078	+	+	J.A. Brito
*M. javanica*	USA, Florida, Pasco County	Peach	CD2063; N16-00078	+	+	J.A. Brito
*M. javanica*	USA, Florida, Polk County	Peach	CD2094; N16-00033	+	+	J.A. Brito
*M. javanica*	USA, Florida, Marion County	Peach	CD2211; N16-00624-3	+	+	J.A. Brito
*M. javanica*	USA, Florida, Marion County	Peach	CD2212; N16-00624-1	+	+	J.A. Brito
*M. javanica*	USA, Florida	Hop	CD2355	+	+	J.A. Brito
*M. javanica*	USA, Florida, Pasco County	Peach	CD2417; N16-00081	+	+	J.A. Brito
*M. javanica*	USA, Florida, Pasco County	Peach	CD2421; N16-00075	+	+	J.A. Brito
*M. javanica*	USA, Florida, Polk County	Peach	CD2428; N16-01289	+	+	J.A. Brito
*M. javanica*	USA, Florida, Polk County	Peach	CD2430; N16-01288	+	+	J.A. Brito
*M. javanica*	USA, Florida, Polk County	Peach	CD2433; N16-01283	+	+	J.A. Brito
*M. javanica*	USA, Florida, Polk County	Peach	CD2443; N16-01268	+	+	J.A. Brito
*M. javanica*	USA, Florida, Pasco County	Peach	CD3049; N16-00084-A	+	+	J.A. Brito
*M. javanica*	USA, Florida, Pasco County	Peach	CD3050; N16-00084-B	+	+	J.A. Brito
*M. javanica*	USA, Florida, Alachua County	Peach	CD3103; N16-01091-3	+	+	J.A. Brito
*M. javanica*	USA, Florida	Mixed roots	CD3105	+	+	J.A. Brito
*M. javanica*	USA, Florida, Polk County	Peach	CD3052; N16-00029-2	+	+	J.A. Brito
*M. javanica*	USA, Florida, Alachua County	Tobacco	CD3099; MJR1-UF	+	+	J.A. Brito
*M. javanica*	USA, UCR collection	Tomato	Isolate 40	+	+	P. Roberts
*M. javanica*	USA, California, Kern County	Tomato	CD3110	+/‒	+	S.A. Subbotin
*M. javanica*	USA, California, Los Angeles County	Vegetables	CD3457	‒	+	S.A. Subbotin
*M. naasi*	Germany	Grasses	CD3381	‒	‒	D. Sturhan
*M. naasi*	USA, California	Grasses	CD2158	‒	‒	S.A. Subbotin
*M. nataliei*	USA, Michigan, Van Buren County	Grapevine	CD3385	‒	‒	S. Álvarez-Ortega
*M. paranaensis*	USA	*Caladium* sp.	CD3510	‒	+	S.A. Subbotin

### Nematode extracts

Several extracts were prepared from: (i) J2s, (ii) J2s with other non-target nematodes, (iii) females and (iv) plant gall tissue with one or more females and egg-masses. The J2 were placed in distilled water on a microscope slide. The nematodes were cut using a dental needle under a stereo microscope and put into 0.2  ml PCR tube with a total volume of 10  μl. This stock crude extract was used to make a series of dilutions sequentially: 1:10, 1:20, 1:50, 1:100, 1:1,000 in water. Crude extract of several hundred non-target, soil free-living (cephalobids, diplogasterids and others) and plant-parasitic nematodes (hoplolaimids, pratylenchids and others) were also obtained by cutting the samples on a microscope slide using a dental needle.

### RPA primer design and testing

Several forward and reverse RPA primers and probes were manually designed in this study. They included: (i) group-specific primers and probes for the tropical RKN complex, which were designed based on polymorphism of the IGS rRNA gene and (ii) species-specific primers and probes to *M. javanica* only, which were designed based on polymorphism of a genome fragment previously characterized by Hu et al. (2011). Primers were synthesized by Integrated DNA Technologies, Inc. (CA, USA). Eleven primer sets were screened in different combinations using the TwistAmp®Basic kit (TwistDx, Cambridge, UK). Reactions were prepared according to the manufacturer’s instructions. Detailed protocol is described by Subbotin and Burbridge (2021). The lyophilized reaction pellets were suspended in 29.5  μl of rehydration buffer, 2.4  μl of each forward and reverse primers (10  μM) (Table 2), 1  μl of DNA template or nematode extract and 12.2  μl of distilled water. For each sample, 2.5  μl of 280  mM magnesium acetate was added to the lid of the tube and the lids were closed carefully. The tubes were inverted 10 to 15 times and briefly centrifuged to initiate reactions simultaneously. Tubes were incubated at 39°C (4  min) in MyBlock Mini Dry Bath (Benchmark Scientific, USA), inverted 10 to 15 times, briefly centrifuged and returned to the incubator block (39°C) for 20  min. Sample tubes were then placed in a freezer to stop the reaction. Amplification products were purified with the QIAquick PCR Purification Kit (Qiagen, USA). Five μl of purified product were run in a 1% TAE buffered agarose gel (100 V, 60  min) and visualized with Gel Green stain (Biotium, USA). Amplification products were directly sequenced by Genewiz (CA, USA).

**Table 2.** RPA primers and probes for amplification of *Meloidogyne* DNA.

Primer or probe	Sequence (5′ – 3′)
*Meloidogyne* spp. from the tropical RKN complex	
Mtrop-RPA-F	ACT TCT AAC AAT CCT TTA TTG ACT CTC G
Meloid-RPA-R	ACA TCA GTT CAG GCA GGA TCA ACC
Meloid-RPA-R-biotin	[Biotin] ACA TCA GTT CAG GCA GGA TCA ACC
Probe-Mtop-RPA-nfo	[FAM]^a^ T GAA TTC TAA AAT TAT CAA TGT AAT CAT TAT [THF]AA TGA CAG CTT AAT TAC CAG [C3-spacer]
Probe-Mtrop-RPA-exo	T GAA TTC TAA AAT TAT CAA TGT AAT CAT [FAM-dT]AT [THF]AA [BHQ1-dT]GA CAG CTT AAT TAC CAG [C3-spacer]
*Meloidogyne javanica*	
Mj-RPA-F	GTG CGC GAT TGA ACT GAG CCC AGA CTG AAA CGA
RPA-mj-R1	AGT GAA GGG CAT CTA TTA GAC ATG GGC
RPA-mj-R1-bio	[Biotin] AGT GAA GGG CAT CTA TTA GAC ATG GGC
Probe-Jav-nfo1	[FAM] AT TTA ATC TAC CGT GTA TGT ATC TAC GTA TAT TGA [THF] GT ATT TGT AAT ATT TAA TTG [C3-spacer]
Probe-Jav-exo1	AT TTA ATC TAC CGT GTA TGT ATC TAC GTA TAT [FAM-dT] GA [THF] G [BHQ1-dT] ATT TGT AAT ATT TAA TTG [C3-spacer]

Note: ^a^FAM—fluorophore, THF—tetrahydrofuran, BHQ—quencher, C3—spacer block.

### Real-time RPA assay

TwistAmp®exo probes for the tropical RKN complex and *M. javanica* specific detection were designed according to the manufacturer’s instructions and were synthesized by Biosearch Technologies, Inc (Petaluma, CA) (Table 2). The real time detection of RPA assay products was accomplished using the TwistAmp^®^exo kit (TwistDx, Cambridge, UK). Detailed protocol is described by Subbotin and Burbridge (2021). The reaction tubes were incubated at 39°C for 5  min, then inverted 10 to 15 times to mix, and briefly centrifuged. The tubes were immediately placed in Applied Biosystems™ QuantStudio™ 7 Flex Real-Time PCR System to incubate at 39°C for 15 min. The fluorescence signal was monitored in real time and measured every 20  sec (cycle) using the FAM channel. A positive control with diluted RPA products of target genes of *M. javanica* and a negative control without any nematode DNA were included in each run. Two or three replicates of each variant across several runs were performed for sensitivity and specificity experiments.

### LF-RPA assay

TwistAmp® nfo probes for the tropical RKN complex and *M. javanica* specific detection were designed according to the manufacturer’s instruction. LF-RPA assay products were accomplished using the TwistAmp® nfo kit (TwistDx, Cambridge, UK). The reaction mixture for each RPA assay was prepared according to manufacturer’s instructions. Detailed protocol is described by Subbotin and Burbridge (2021). The reaction tubes were incubated at 39°C for 4  min, then inverted 10 to 15 times to mix, briefly centrifuged and returned to the incubator block at 39°C for 16  min. Tubes were placed in freezer to stop reaction. For visual analysis with the Milenia® Genline Hybridetect-1 strips (Milenia Biotec GmbH, Germany), a testing solution containing 48  μl of HybriDetect assay buffer and 12  μl of the sample RPA product was prepared in a 0.5  ml PCR tube. Ten μl of the testing solution was placed directly onto the sample area of the dipstick. Dipsticks was placed upright into 100 μl of assay buffer and visual results were observed within 5 min. The amplification product was indicated by the development of a colored test line and/or a control line to confirm that the system worked properly. Two replicates of each variant were performed for sensitivity and specificity experiments.

## Results

### RPA diagnostic assay for the tropical RKN complex

#### RPA detection assay with visualization on gel

The group-specific forward Mtrop-RPA-F primer was designed and tested with the reverse Meloid-RPA-R primer proposed by Subbotin (2019). This primer set produced a band of ~ 313 bp in a length in RPA reactions using TwistAmp^®^Basic kit with nematode extracts and DNA from species belonging to the tropical RKN complex. No band of this size was observed on gels with other species from non-tropical RKN complex. The sequences of primers and probes used for the assays are listed in Table 2.

### Real-time RPA detection assays

**Figure 1:**
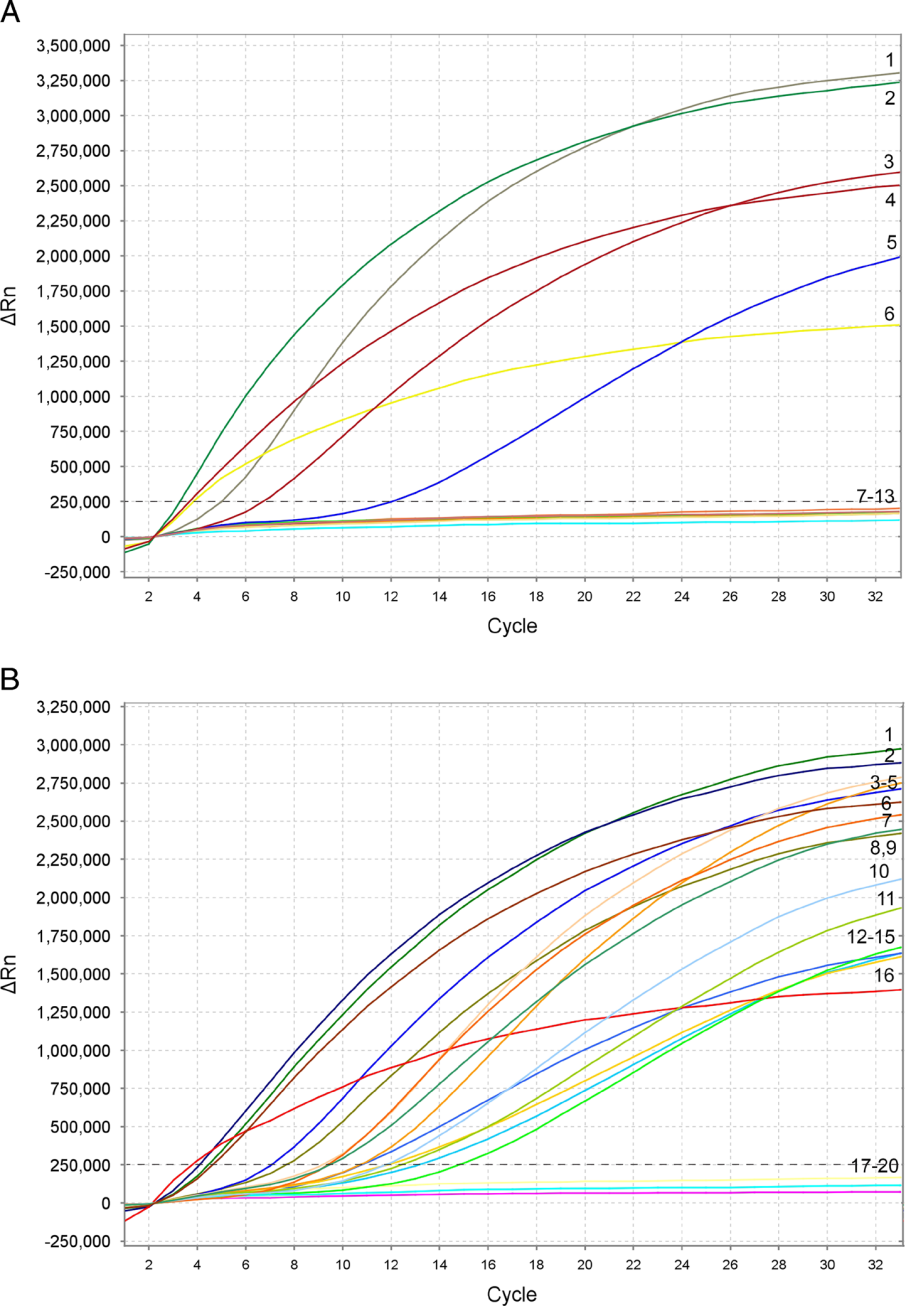
RPA assay using real-time fluorescent detection with examples of amplification plots for *Meloidogyne* spp. from the tropical group. (A) Specificity assay with *Meloidogyne* samples. Amplification curve: 1—*M. floridensis* (CD3111); 2—*M. incognita* (CD3038); 3—*M. javanica* (CD3050); 4—*M. javanica* (isolate 40); 5—*M. javanica* (CD3110); 6—*M. javanica* (positive control); 7—*M. christiei* (CD1471); 8—*M. hapla* (VW9); 9—*M. naasi* (CD2158); 10—*M. naasi* (CD3381); 11—*M. nataliei* (CD3385); 12—*M. baetica* (CD3382); 13—negative control. (B) Sensitivity assay with a crude J2 extract of *M. javanica* (isolate 40). Dilution series: 1 J2 per tube—1, 2 and 6; 1/10 J2 per tube—5, 7 and 9; 1/20 J2 per tube—3, 8 and 15; 1/50 J2 per tube—11, 13 and 14; 1/100 J2 per tube—4, 10 and 12; 1/1000 J2 per tube—17, 18 and 19; positive control—16; negative control—20. The vertical line on a graph: fluorescence ∆Rn. ∆Rn is calculated at each cycle as ∆Rn (cycle) = Rn (cycle)-Rn (baseline), where Rn = normalized reporter. The horizontal line on a graph: cycles, each cycle = 20 sec.

Based on the results of ten experimental runs, the threshold level for reliable tropical group detection was established as equal to 18 cycles (~6  min) with a baseline of 250,000 (∆Rn) fluorescence using the TwistAmp^®^exo kit on the Applied Biosystems™QuantStudio™7 Flex Real-Time PCR System (Fig. 1). Samples that produced an exponential amplification curve above the threshold were considered as positive for the tropical RKN complex and below the threshold were considered as negative. Detections of the tropical RKN complex species were confirmed with all samples containing extracts and DNA from these species: *M. arenaria, M. floridensis, M. hispanica, M. incognita, M. javanica* and *M. paranaensis* (Fig. 1A). This assay did not detect *M. haplanaria* and *M. enterelobii* (data not shown).

Sensitivity assay tests were run with serial dilutions of crude J2 extractions. The reliable detection level was estimated at 1/100 of a J2 per reaction tube (Fig. 1B). The results also showed a reliable detection of a single J2 with DNA extracted from 10 and 20 non-target nematodes and extracts from root galls containing females with eggs-masses belonging to the tropical RKN complex (data not shown).

### LF-RPA detection assay

**Figure 2:**
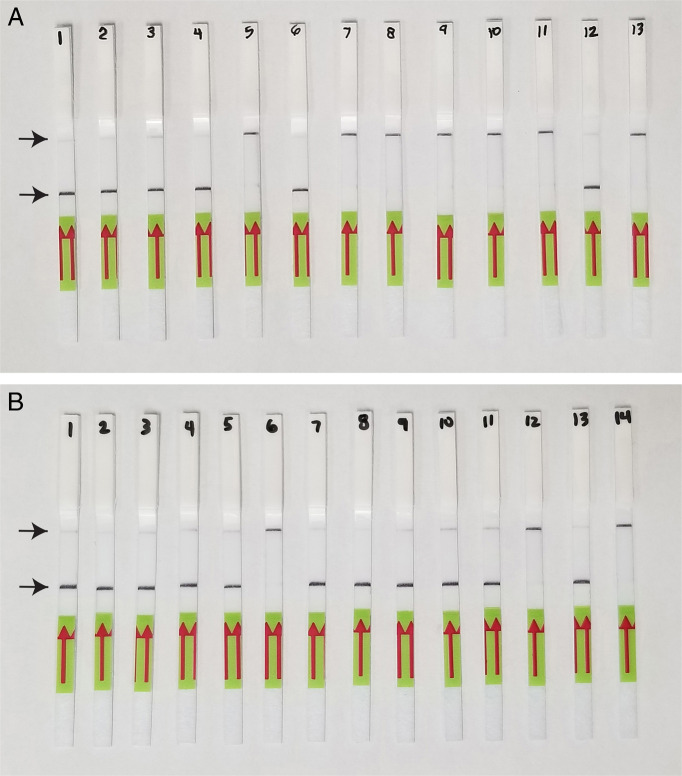
Lateral flow recombinase polymerase amplification (LF-RPA) assay with examples of strips. (A) Specificity test. Strip: 1—*M. javanica* (isolate 40); 2—*M. javanica* (CD3050); 3—*M. javanica* (CD3110); 4—*M. incognita* (CD3038); 5—*M. hapla* (VW9); 6—*M. floridensis* (CD3111); 7—*M. christiei* (CD1471); 8—*M. naasi* (CD2158); 9—*M. naasi* (CD3381); 10—*M. baetica* (CD3382); 11—*M. nataliei* (CD3385); 12—*M. arenaria* (positive control); 13—negative control. (B) Sensitivity test. *M. javanica* (isolate 40); Strip:1 and 7—1 J2 per tube; 2 and 8—1/10 J2 per tube; 3 and 9—1/20 J2 per tube; 4 and 10—1/50 J2 per tube; 5 and 11—1/100 J2 per tube; 6 and 12—1/1000 J2 per tube; 13—*M. arenaria* (positive control); 14—negative control.

Lateral flow detection of RPA products using the TwistAmp®exo kit also showed specific and sensitive results. Positive test lines on the LF strips were observed for all the tropical RKN complex samples (*M. arenaria, M. floridensis, M. hispanica, M. incognita, M. javanica* and *M. paranaensis*), whereas samples with other nematode species showed only a control line (Fig. 2A). The results of RPA assays showed reliable detection with a sensitivity of 1/100 of a J2 (Fig. 2B) and 1/10 of a female (data not shown) in reaction tubes. The results of RPA assay also showed a reliable detection of a single J2 with DNA extracted from10 and 20 non-target nematodes (data not shown) and extracts from root galls containing females with eggs-masses belonging to the tropical RKN complex (data not shown).

### RPA diagnostic assay for *Meloidogyne javanica*

#### RPA detection assay with visualization on gel

The genome fragment of *M. javanica* with GenBank accession number JN005834 were aligned with similar genome fragments of this species (RCFK01023427; CEWN0101050) and other RKN species (RCFN01005875—*M. floridensis*; RCFL01003500—*M. incognita*). Eleven primers were designed based on species polymorphism for *M. javanica* and then screened for the best performance in RPA with the TwistAmp®Basic kit. The species-specific forward Mj-RPA-F primer proposed by Ju et al. (2019) and the species-specific reverse RPA-mj-R1 primer were found to produce a species-specific band for *M. javanica* with a length of 213 bp and had no cross-reactions with other RKN. The sequences of primers and probes used for the assays are listed in Table 2.

#### Real-time RPA detection assays

**Figure 3:**
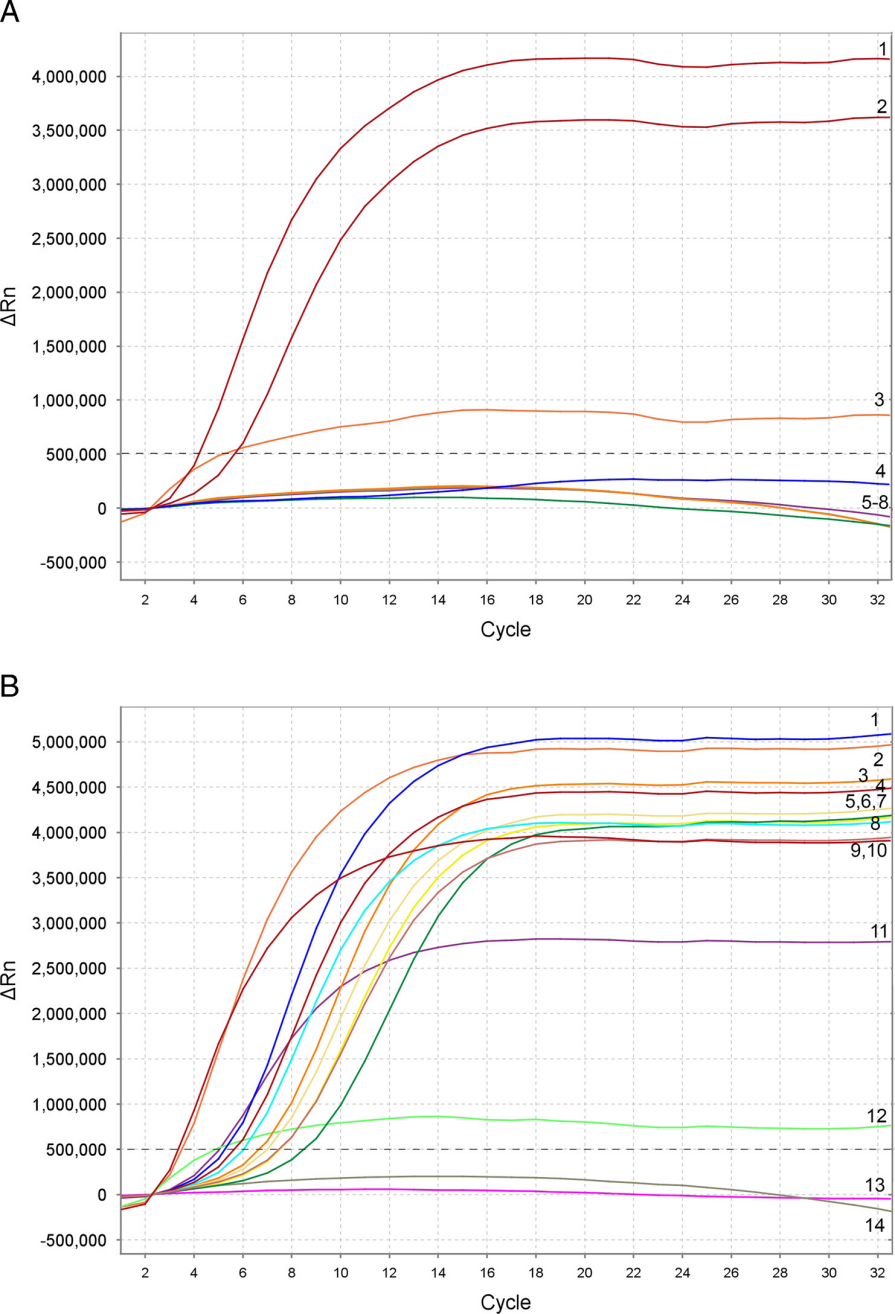
RPA assay using real-time fluorescent detection with examples of amplification plots for *M. javanica*. (A) Specificity assay with *Meloidogyne* samples. Amplification curve: 1—*M. javanica* (CD3050); 2—*M. javanica* (isolate 40); 3—*M. javanica* (positive control); 4—*M. javanica* (CD3110); 5—*M. incognita* (CD3038); 6—*M. floridensis* (CD3111); 7—*M. hapla* (VW9); 8—negative control. (B) Sensitivity assay with a crude J2 extract of *M. javanica* (isolate 40). Dilution series: 1/10 J2 per tube—4 and 11; 1/20 J2 per tube—1 and 9; 1/50 J2 per tube—5 and 8; 1/100 J2 per tube—3 and 7; 1/1000 J2 per tube—6 and 14; positive control—2 and 10; negative control—13. The vertical line on a graph: fluorescence ∆Rn. ∆Rn is calculated at each cycle as ∆Rn (cycle) = Rn (cycle)-Rn (baseline), where Rn = normalized reporter. The horizontal line on a graph: cycles, each cycle = 20 sec.

Based on the results of twelve experimental runs, the threshold level for reliable *M. javanica* detection was established as approximately to 10 and up to 16 cycles (~3.5–5  min) with a baseline of 500,000 (∆Rn) fluorescence using the TwistAmp^®^exo kit on the Applied Biosystems™QuantStudio™7 Flex Real-Time PCR System (Fig. 3). Samples that produced an exponential amplification curve above the threshold were considered as positive for *M. javanica* and below the threshold were considered as negative. Species-specific detection was confirmed with most samples containing extracts or DNA of this species, except for the two samples originated from California (Table 1; Fig. 3A) which gave negative results in some replicates. Additional testing revealed that this sample cannot be consistently identified as *M. javanica* using the present RPA assay.

The RPA assay was tested for specificity using DNA extracted from several RKN: *Meloidogyne arenaria, M. baetica, M. christiei, M. enterelobii, M. hapla, M. haplanaria, M. hispanica, M. floridensis, M. incognita, M. javanica, M. naasi, M. nataliei* and *M. paranaensis*. The RPA results using real-time fluorescent detection showed specificity only to *M. javanica* (Fig. 3A).

The sensitivity assay tests were run with serial dilutions of crude J2 extractions. The reliable detection level of *M. javanica* was estimated at 1/100 of a J2 per a RPA reaction tube although the detection level was 1/1000 of a J2 in some replicates (Fig. 3B).

The detection of a single J2 for *M. javanica* was confirmed in the presence of background crude extracts from 10 and 20 non-target nematodes. No decrease in fluorescent signals was observed between these variants (data not shown).

#### LF-RPA assay

**Figure 4:**
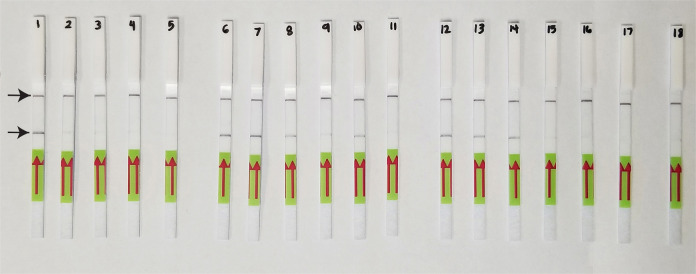
Lateral flow recombinase polymerase amplification (LF-RPA) assay with examples of strips from specificity and sensitivity tests. Strip: 1—*M. javanica* (CD3050); 2—*M. javanica* (CD3110); 3—*M. incognita* (CD3038); 4—*M. hapla* (VW9); 5—*M. floridensis* (CD3111), 6–17—*M. javanica* (isolate 40); 6 and 12—1 J2 per tube; 7 and 13—1/10 J2 per tube; 8 and 14—1/20 J2 per tube; 9 and 15—1/50 J2 per tube; 10 and 16—1/100 J2 per tube; 11 and 17—1/1000 J2 per tube; 18: negative control.

Lateral flow detection of RPA products also showed specific and sensitive results. Positive test lines on the LF strips were observed for most *M. javanica* samples, except for two samples from California. Samples with other nematode species showed only a control line (Fig. 4). The results of RPA assays showed reliable detection with a sensitivity of 1/20 of a J2 (Fig. 4). The detection of J2 for *M. javanica* was confirmed in the presence of background crude extract from 10 and 20 non-target nematodes (data not shown).

## Discussion

The RKN control programs aim to reduce impact and distribution of these pests in agricultural fields and nurseries. However, effective control and eventual elimination of these nematodes needs high sensitive diagnostic tests. In this study we developed a rapid, reliable and affortable method of detection for RKN belonging to the tropical complex using RPA detection technology. The entire detection process for real-time RPA assay can be completed within approximately 16 min, including 4 min for crude nematode extract preparation, 11 min (5 + 6) for the RPA reaction with 1 min for mixing and centrifugation of tubes. The entire detection process for the LF-RPA assay can be completed within approximately 30 min, including 4 min for crude nematode extract preparation, 20 min (4 + 16) for the RPA reaction, 1 min for mixing and centrifugation of tubes, and 5 min for visual detection on the LF strips. This calculation does not include the time for preparation of the RPA reaction mixture.

In this study we developed the tropical RKN complex group-specific RPA assay, which allowed making preliminary screening to detect the presence of any species from this group in samples. It should be noticed that J2 of the RKN could be easily misidentified with Tylenchidae and other nematode juveniles and this assay can help to make a correct identification. To develop species-specific RKN RPA assay in the lab, several species-specific primers for *M. incognita, M. arenaria* and *M. javanica* were tested, but only species-specific primers of *M. javanica* showed species-specific amplification and the species-specific assay was only developed to this species in the frame of this project.

For desinging the species-specific *M. javanica* RPA primers and probe we used a genome fragment firstly proposed by Randig et al. (2002) and then applied for RPA assays by Ju et al. (2019) and Chi et al. (2020). In our RPA assay the forward species-specific RPA primer proposed by Ju et al. (2019) and a new reverse primer were used. This primer set was species-specific for *M. javanica* only. Although, the test specifically identified a wide range of *M. javanica* samples, it did not detect two populations of *M. javanica* from California. Further testing of more *M. javanica* populations as well as testing with low number of nematodes in samples is needed to further assess the performance of this assay.

Advantages and disadvantages of the RPA method for nematode diagnostics are discussed in our publications (Subbotin, 2019; Subbotin and Burbridge, 2021). Sensitivity of the present assays is comparable with those already developed by Ju et al. (2019) and Chi et al. (2020). However, our assays do not requre DNA extraction and allow for detection of nematodes in plant tissues and in a mixure with other nematodes. RPA assay has great potential for diagnosing the RKN in the lab, field or in areas with a minimal laboratory infrastructure.
